# Postmastectomy Pain Syndrome: A Narrative Review

**DOI:** 10.7759/cureus.47384

**Published:** 2023-10-20

**Authors:** Sajad Ahmad Salati, Lamees Alsulaim, Mariyyah H Alharbi, Norah H Alharbi, Thana M Alsenaid, Shoug A Alaodah, Abdulsalam S Alsuhaibani, Khalid A Albaqami

**Affiliations:** 1 General Surgery, Unaizah College of Medicine & Medical Sciences, Qassim University, Qassim, SAU; 2 Surgery, Unaizah College of Medicine & Medical Sciences, Qassim University, Qassim, SAU; 3 College of Medicine, Unaizah College of Medicine & Medical Sciences, Qassim University, Qassim, SAU

**Keywords:** neuroma, neuromodulation, gabapentinoids, neuropathy, intercostobrachial nerve, antidepressants, postmastectomy pain syndrome, mastectomy

## Abstract

Postmastectomy pain syndrome is a very common disorder in breast cancer survivors. The impact on the quality of patients’ lives is significantly adverse. The precise pathophysiology has not been determined as yet though various risk factors have been identified that make the patient vulnerable. Required preoperative work includes the identification and possible elimination of risk factors. Treatment is multidisciplinary involving surgical and non-surgical modalities. There is a great scope of research in this field.

## Introduction and background

Breast cancer is one of the most commonly diagnosed cancers among women and the leading cause of cancer-related death among them. The treatment modalities and the outcome of breast cancer have witnessed substantial improvement over the last few decades. However, over time, the adverse effects of treatment have also become increasingly obvious [1]. The treatment of this cancer depends on the stage at which it presents, and the primary treatment of nearly 37-40% of women with breast cancer is surgical resection that includes different variants of mastectomy with or without reconstruction like modified radical mastectomy, skin-sparing mastectomy, simple mastectomy, etc. [2]. After surgical procedures, a number of complications may arise. Postmastectomy pain syndrome (PMPS) is a common postoperative complication that affects 20-68% of women who have undergone mastectomy [3,4]. Wood first described persistent pain after mastectomy in 1978 [5], but despite reports of a high incidence of this complication, research on PMPS seems to be lacking. There is no clear consensus on the specific definition of PMPS, nor have the etiology and pathogenesis of PMPS been determined. PMPS has been documented to be extraordinarily burdensome and to have a detrimental effect on overall functional ability and quality of life (QOL) in women who survive long after mastectomy [6].

In light of the foregoing, this article has been compiled to review the current understanding of PMPS with an emphasis on epidemiology, etiopathogenesis, presentation, risk factors, prevention, and treatment options. The primary purpose of this article is to raise awareness of the disorder among medical professionals and encourage more research on the subject. 

## Review

Methods

**Figure 1:**
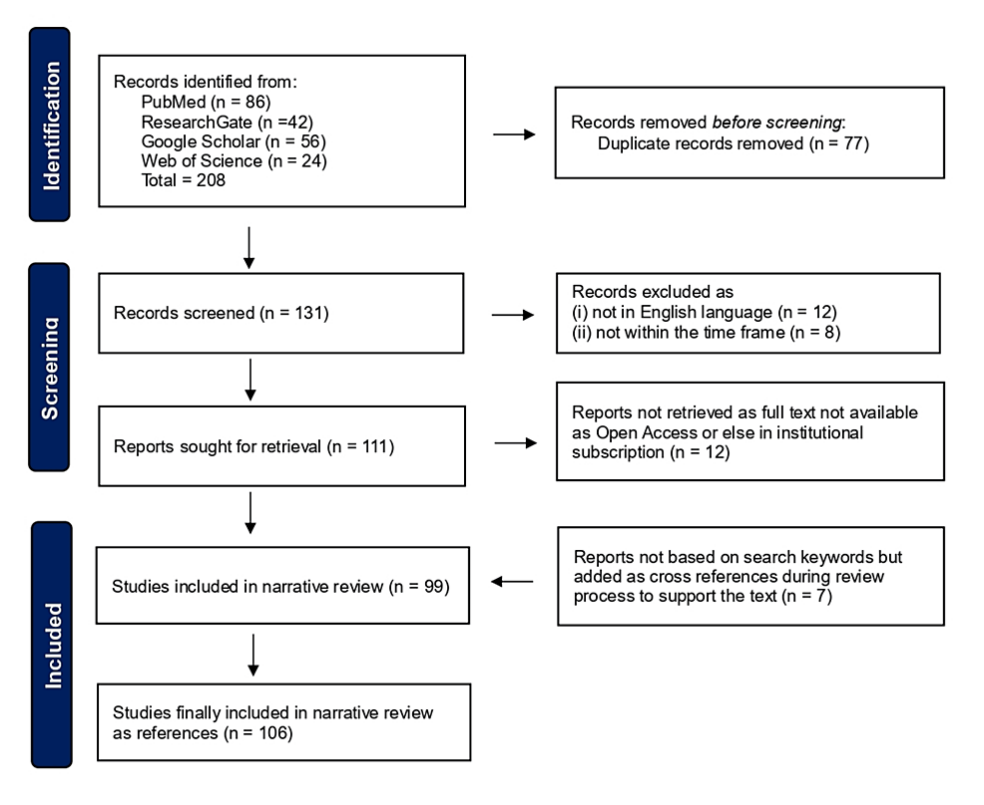
Flowchart showing the selection process. Image credit: Sajad Ahmad Salati (Author)

The databases of PubMed, ResearchGate, Google Scholar, and Web of Science were searched for observational studies, reviews, and case reports pertaining to PMPS. The last four authors (KAA, ASA, SAA, TMA) manually searched a single database each, and after comparing the results, duplicate entries were removed (Figure 1). No automation tools were utilized. The search was based on the keywords: ‘Postmastectomy pain syndrome’, ‘Postmastectomy neuropathy, ‘Postmastectomy pain’, and ‘Chronic mastectomy pain’. Publications written between 2000 and 2023 in the English language were included; articles published in other languages were excluded (Figure 1). Additionally, after analyzing the included papers, certain cross-references were accommodated if they provided historical context or else provided evidence that articles within the above timeframe did not provide.

Epidemiology

According to reports in the literature, 20-68% of breast cancer survivors suffer from some degree of PMPS [7,8]. In a Danish study by Vilholm et al., the prevalence of PMPS was reported to be 24% in 258 surgically treated breast cancer patients [9]. It was determined that the odds ratio for developing PMPS was 2.9. In a study carried out in China by Gong et al., 560 (28.2%) cases of patients who had undergone surgical treatment for breast cancer experienced PMPS [10]. Gartner et al. found that of the 3253 patients, a total of 1543 (47%) reported PMPS, of whom 201 (13%) had severe pain, 595 (39%) had moderate pain, and 733 (48%) had light pain [11]. Couceiro et al. [12] found PMPS in 44.4% of patients, and in the series by Alves Nogueira Fabro et al. [13], 52% of the enrolled 174 breast cancer survivors suffered from PMPS.

Pathophysiology

The precise pathophysiology has not yet been determined. Although other explanations have been put forward, intercostobrachial nerve neuralgia is currently considered to be the most likely cause of PMPS. The intercostobrachial nerve is the cutaneous lateral branch of the second intercostal nerve that emerges through the serratus anterior muscle, providing sensation to the axilla and the internal portion of the upper arm.

During axillary dissection, the intercostobrachial nerve may get damaged to various degrees by stretching or compression during retraction (neuropraxia, axonotmesis) or by frank transection (neurotmesis). There is a possibility of indirect nerve injury intraoperatively or postoperatively. Intraoperatively, poor arm positioning can stress and compress the nerve, and postoperatively, hematoma, seroma, and scarring can result in stretch and compression injuries.

The nerve injury is followed by ectopic neural activity originating at the nerve injury site and dorsal root ganglion, resulting in increased sensitivity to chemical or mechanical stimuli and a subsequent increased perception of pain [14-16]. Other nerves related to PMPS include the medial and lateral pectoral, thoracodorsal, long thoracic, and intercostal nerves [17].

Etiology

**Figure 2:**
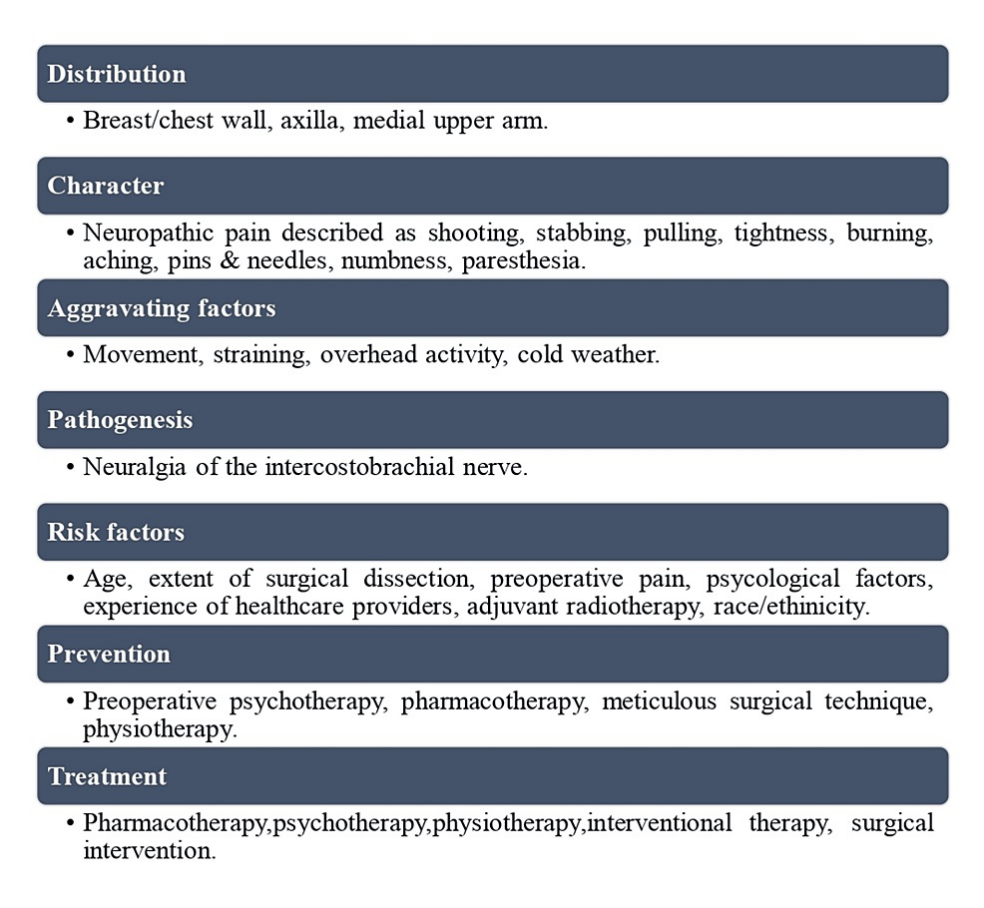
Common characteristics of persistent postmastectomy syndrome (PMPS). Image Credit: Sajad Ahmad Salati (Author)

Although the exact etiopathogeneses are still unclear, many risk factors (Figure 2) that may play a part in the emergence of PMPS have been put forth [18,19].

Age < 40 Years

It has been discovered that younger age groups pose a risk. The reason for this association is not clear, though several possibilities have been suggested, including a higher histopathological tumor grade in younger patients with a requirement for adjuvant chemotherapy, generally decreased pain receptor sensitivity in older patients, and differences in estrogen receptor status [20-22]. In a series by Alves Nogueira Fabro et al., the mean age of 174 studied patients was 58 years, and it was found that younger women (<40 years) show a significantly increased risk of PMPS (relative risk (RR) = 5.23, 95%CI: 1.11-24.64) [13].

Type of Surgery

The type of surgery has been linked to the prevalence of PMPS, and it has been discovered that, in contrast to breast conservation procedures and sentinel lymph node biopsy, the more invasive breast operations like the different variants of mastectomy (e.g., radical mastectomy, modified radical mastectomy, skin-sparing mastectomy, simple mastectomy, etc.) and axillary lymph node dissection are more likely to damage sensory nerves in advanced cancers [23]. According to Alves Nogueira Fabro et al., patients who underwent axillary lymph node dissection (with more than 15 lymph nodes removed) had a noticeably higher chance of developing PMPS (RR = 2.01, 95%CI: 1.08-3.75) [13].

Psychosocial Factors 

Numerous significant studies demonstrate that various psychological factors play a significant role in raising PMPS sensitivity. Miaskowski et al.'s longitudinal study of 410 cancer survivors revealed a substantial correlation between preoperative anxiety and depression and the eventual development of moderate-to-severe PMPS [24]. Belfer et al., in a cross-sectional cohort study, followed 611 patients for three years and concluded that anxiety, catastrophizing, sleep disturbance, and somatization were independently associated with PMPS [25].

A study by Meretoja et al. enrolled 970 patients and documented associations of both anxiety and depression with subsequent PPMP [26]. Ghadimi et al. investigated the possible relationship of PMPS with clinical and psychological variables and concluded that loneliness may be a significant risk factor [27].

Several epidemiological studies have suggested that body mass index (BMI) may be potentially associated with PMPS [26,28-30]. In order to assess the relationship between BMI and PMPS, Ding et al. first conducted a prospective study [29]. Then, using a meta-analysis, they synthesized the evidence from eight observational studies that were found by searching PubMed and Embase until February 2017. They concluded that BMI may be positively correlated with the risk of PMPS.

Along similar lines, the potential association of smoking with PMPS has also been analyzed in some studies. Aliyev and Asik, in a prospective study, observed that PMPS developed in 27 (90%) of 30 patients who smoked preoperatively and, hence, a statistically significant relationship was established between smoking and CPPS (p<0.001) [31]. Sipila et al. found that smoking induces changes at the neuronal level in the nervous system that stay for a long time and contribute to the emergence of PMPS [32]. Nicotine in tobacco products has been found to play a critical role in pain-related pathophysiology. It has proven acute analgesic effects but the smokers get exposed to it on long-term exposure, which leads to the development of tolerance and increased pain sensitivity due to desensitization of nicotinic acetylcholine receptors and neuronal plastic changes. Yamaguchi et al. drafted a consensus statement on smoking cessation in patients with pain of any origin and summarized the available evidence on the impact of smoking on pain [33]. They stressed that smoking cessation interventions not only reduce pain but also improve the outcomes of underlying pain-causing conditions and reduce the risks of tobacco-related disorders.

Preoperative Pain

It has been discovered that preoperative pain at the surgical site increases the risk of PPMP. Although the exact processes are unknown, it has been hypothesized that pain sensitivity and/or central sensitization may increase the susceptibility to persistent pain following surgery [34]. In their study, Roth et al. discovered that over half of the individuals had experienced preoperative pain to some extent [35]. They came to the conclusion that chronic pain following breast reconstruction might not always be related to pain from the operation.

Surgical Department Patient Turnover 

Patients treated at low-volume healthcare facilities that undertake fewer than 100 mastectomy operations of different variants annually have a noticeably greater incidence of PMPS than patients operated on at surgical units that perform more than 100 mastectomies annually. According to Tasmuth et al., high turnover improves experience and aids surgical teams in honing their skills [36]. The more experienced teams frequently employ safer patient positioning and more precise surgical techniques, thereby ensuring that the dissection is carried out with greater care, minimizing the possibility of nerve injury during the procedure, and avoiding issues like hematoma, seroma, and dense scarring that could potentially result in stretch or compression of the nerves.

This is a trend that has been witnessed with many other surgical procedures. For example, Gorbea et al. found lesser overall perioperative complications in the hands of otolaryngologists who perform a high volume of total thyroidectomy than those with less volume, and the risk of temporary tracheostomy was particularly higher among low-volume surgeons [37]. These findings are consistent with previous studies of the effect of thyroidectomy volume on surgical complications. Hauch et al. also found that higher surgeon volume is associated with improved patient outcomes and fewer complications [38].

Sociodemographic Factors, Race/Ethnicity

According to data, race or ethnicity and sociodemographic characteristics affect the risk of developing PMPS [19]. Minorities, such as Latinas and African American women, exhibited a higher prevalence and intensity of PMPS, according to Eversley et al. [39]. There are a number of explanations given [11], including the fact that patients from ethnic minorities often receive diagnoses at later stages of the disease, necessitating extensive surgical procedures and more aggressive neoadjuvant and adjuvant therapies (such as chemotherapy and radiation therapy).

Chemotherapy 

The breast cancer management has witnessed an increased use of systemic agents, including taxanes, platinum agents, and vinca alkaloids as part of chemotherapy regimens and aromatase inhibitors (AIs) as hormonal therapy. Many studies have found a potential relationship with the development of PMPS and specific terms like persistent pain in breast cancer treated with chemotherapy (PPBCC) and persistent pain in breast cancer hormonal therapy (PPBCHT) have been proposed [40,41].

Postoperative Factors

A major risk factor consistently linked to the emergence of PMPS is the presence of severe postoperative pain. In a study by Bruce et al. involving 362 postmastectomy patients, it was discovered that higher postoperative pain levels were associated with higher odds of PMPS at four months (OR = 1.34, 95%CI = 1.12-1.60) and at nine months (OR =1.17, 95%CI = 1.00-1.37) [42]. Andersen et al. discovered that higher postoperative pain levels raised the likelihood of moderate-to-severe PMPS after one year [43].

Radiation Therapy

PMPS risk has been observed to be greatly enhanced when neoadjuvant or adjuvant radiation therapy is delivered over the chest wall and/or axilla as a part of multimodality breast cancer treatment [44,45]. This is related to tissue fibrosis, which results in nerve entrapment and restricted shoulder joint movement [11]. Furthermore, skin reactions and lymphedema may add to the discomfort levels [45]. According to Gartner et al., adjuvant radiation was linked to a higher risk of PMPS (OR =1.5, 95%CI: 1.08-2.07, p = 0.03) [11].

Definition and clinical presentation 

There is an ambiguity surrounding the definition of PMPS in literature, and several definitions have been used. PMPS is most commonly defined as a chronic pain that lasts beyond the normal healing time, when all other potential causes of pain, such as infection or recurrence, have been eliminated [46,47]. Vilholm et al. [9] defined PMPS as neuropathic pain with intensity greater than 4 on a 10-point numeric scale, lasting longer than six months following surgery, while Couceiro et al. [12] label pain persisting for longer than three months as PMPS.

Technically, the term PMPS is rather misleading, as the syndrome has been reported to occur even after breast-conserving surgical operations [13,48]. The pain is often located in the axilla, the shoulder, the arm, or the chest wall or breast region. PMPS is often described as a typical neuropathic pain (Figure 2) characterized by burning, painful cold, electrical shock sensations, stinging, numbness, tingling, stabbing, pulling, or pins and needles sensations [46,49]. Pain may be deep, blunt in character, and evoked by pressure. In a series by Kakati et al., pain was located in 120 patients over the anterior chest wall (41.8%), axilla (32.6%), and medial upper arm (25.6%) [4]. PMPS gets exacerbated by various factors, which include movement, straining, overhead activity, and exposure to cold [50].

The onset of pain is most likely to occur after surgery, though there may also be a new onset of symptoms after many months following adjuvant therapy such as chemotherapy or radiation therapy [51]. Kakati et al. prospectively followed up 120 women after mastectomy to identify the incidence and severity of PMPS along with its impact on QOL in Indian patients, by using the Brief Pain Inventory (BPI) questionnaire and the QOL questionnaire (QLQ) by the European Organization for Research and Treatment of Cancer (EORTC-QLQ 30) [4]. They found that 35.8% of patients suffered from PMPS at six months and the character of pain was described as dull aching by about 49%. About 56% of patients had mild-intensity pain, and they were attempting to manage and accept it as an inevitable component of their treatment. Macdonald et al. assessed the long-term outcome of PMPS at 7-12 years postoperatively and found that 52% of the patients continued to experience PMPS at a mean of nine years after surgery [3].

To prevent discrepancies in clinical as well as research settings, Waltho and Rockwell tried to establish a consensus for defining PMPS by identifying the various elements and proposing a new and complete definition [46]. Accordingly, they defined PMPS as a pain that occurs after any breast surgery, which is of at least moderate severity, possesses neuropathic qualities, is located in the ipsilateral breast/chest wall, axilla, and/or arm, lasts at least six months, occurs at least 50% of the time, and may be exacerbated by movements of the shoulder girdle.

Impact on QOL

PMPS has been found to exert a significantly negative impact on the overall QOL, even in patients in whom its severity is mild. PMPS may limit the range of motion (ROM) of the upper extremities, which would negatively impact the capacity to perform daily tasks and even basic occupational tasks. Furthermore, PMPS has been found to take a significant toll on mental health, emotional well-being, and social relationships [22,52]. 

Ferreira et al. assessed the impact of PMPS on the lives of 30 breast cancer survivors using the BPI and found that pain exerts a negative impact on mood, normal work, and sleep [53]. Caffo et al. explored the physical well-being, physical autonomy, relational life, and psychological well-being of patients undergoing surgical treatment for breast cancer and found that the patients who developed PMPS had significantly worse QOL scores on all of the subscales than those who did not develop PMPS [6]. Fontes et al. studied the impact of PMPS on patients' sleep quality and found a significant association between PMPD and deterioration in sleep quality during the first year of follow-up, more pronounced among those with good sleep quality (Pittsburgh Sleep Quality Index (PSQI) 5) than those with poor sleep quality at baseline (PSQI >5) [54]. Daytime dysfunction and sleep duration were found to be the most impaired components.

Bibi et al. employed descriptive correlational research design, using the BPI Short Form (BPI-SF) to assess PMPS and the Functional Assessment of Cancer Therapy-Breast (FACT-B) to measure the health-related QOL and found that there was a significant negative impact on the health-related QOL, particularly on the physical well-being [55]. Burckhardt et al. compared and contrasted the pain characteristics, symptom impact, health status, and QOL of post-breast cancer surgery patients with regional chronic pain with those with widespread chronic pain and concluded that the women who experienced widespread pain had significantly more severity of pain and pain impact, and lower physical health status than those with regional pain [56]. 

Prevention of PMPS

Patients with strong risk factors for PMPS need to be identified, and proper efforts need to be made for its prevention through the use of multimodal care involving psychotherapy, proper surgical technique, physiotherapy, and peripherally targeted or centrally acting pharmacotherapy [57]. Multiple studies have found that active preoperative psychological counseling and social support can reduce the severity of PMPS [58,59]. Hence, caregivers need to be aware of and make available interventions that strengthen resilience and provide social support. Liukas et al. retrospectively reviewed 101 electronic health records of patients with breast cancer, and 337 different expressions describing psychosocial factors associated with postoperative persistent pain were identified, which included psychological strength and resilience, social factors, emotional factors, anxiety, sleep-related factors, and depression [60]. They found that although psychosocial factors associated with PMPS were placed in the electronic health records, documentation about such factors was not found in all patients' records, nor was the documentation done in a systematic manner. The study concluded that there is potential to use electronic health records as an information source in the development of decision-support system algorithms to better support nurses in the identification of patients at risk of developing PMPS, provided quality standards are set and applied for comprehensive documentation.

During operations on the breast, attempts are to be made to minimize the damage to the pectoralis major, pectoralis minor, and the surrounding fascia and if axillary exploration is undertaken, care should be taken to avoid injury to the intercostobrachial nerve. Studies suggest that nerve block during surgery may prevent PMPS, and a study by O’Brien et al. showed promising results with targeted muscle reinnervation, wherein transection of the lateral cutaneous intercostal branch was identified immediately and coaptation of the intercostal nerve to the target motor muscle was accomplished [61]. Gatherwright and Knackstedt have reported the use of cadaveric nerve grafts for targeted reinnervation of damaged intercostal nerves [62]. In their randomized control trial, Fujii et al. studied pectoral nerve-2 (PECS 2) block vs. serratus plane block for chronic pain after mastectomy [63]. The study found that a pectoralis nerve block decreased the rate of moderate to severe chronic pain at six months postoperatively compared with a serratus anterior block.

In the postoperative phase, early commencement of physiotherapy can play a helpful role in maintaining glenohumeral and scapular chest movement, strength, and normal neuromuscular recruitment patterns, thereby minimizing upper limb dysfunction and PMPS [64]. 

Pharmacologic agents, including gabapentinoids, memantine, and nefopam, administered in the perioperative period have been found to be safe and efficacious in reducing acute pain and the subsequent development of PMPS [65,66]. In their randomized control trial, Fassoulaki et al. evaluated the impact of multimodal analgesia on acute and chronic pain after breast surgery for cancer and found that by combining perioperative gabapentin and local anesthesia, a significant decrease in PMPS can be achieved over the long term, thereby supporting the importance of a multimodal approach to the prevention of PMPS [67].

Amr and Yousef prospectively investigated the analgesic efficacy of venlafaxine and gabapentin on pain associated with mastectomy for cancer [68]. A total of 150 patients scheduled for either partial or radical mastectomy with axillary dissection were randomized to receive extended-release venlafaxine (37.5 mg/d), gabapentin 300 mg/d, or placebo for 10 days starting the night before the operation. It was found that at six months post the operation, the incidence of chronic pain, its intensity, and the need for analgesics were reduced in venlafaxine compared to gabapentin and the placebo group, but the burning type of pain was more frequent in the control groups than in gabapentin.

Memantine is an NMDA receptor antagonist. Morel et al. carried out a randomized, pilot clinical trial enrolling 40 women undergoing mastectomy [69]. The aim was to evaluate whether memantine administered before and after mastectomy could prevent the development of PMPS and the impairment of cognition. Accordingly, memantine (5-20 mg/day; n = 20) or placebo (n = 20) was administered for four weeks, starting two weeks before surgery. The study found that, at three months, patients receiving memantine showed a significant difference in postmastectomy pain intensity, less rescue analgesia, and a better emotional state as compared to the placebo group.

Nefopam is a non-opioid, non-steroidal benzoxazocine analgesic that is believed to act by inhibiting the reuptake of serotonin, norepinephrine, and dopamine. Additionally, it modulates the calcium and sodium channels in the glutamatergic pathway, thereby decreasing the activation of N-methyl-D-aspartate (NMDA) receptors. Na et al. prospectively evaluated the efficacy of nefopam on acute and chronic postoperative pain after breast cancer surgery [70]. The patients were assigned to the nefopam (n = 41) or control (n = 42) groups. Before the start of the operation, 20 mg of nefopam was administered to the patients in the nefopam group, and normal saline was used in the control group. The study concluded that preventive nefopam reduced acute postoperative pain with reduced use of rescue analgesic drugs and contributed to a reduced incidence of PMPS at three months.

Proper control and alleviation of pain in the postoperative phase is important in other ways too, as studies have shown that improperly controlled pain increases hospitalization.

Treatment 

PMPS involves chronic pain as well as psychosocial and functional disruption, and hence, a multidisciplinary approach can address all these domains. While adopting a multidisciplinary approach, the team may comprise a plastic surgeon, a pain specialist, a physical therapist, an oncologist, a radiologist, and a clinical psychologist. An important step in management is the exclusion of other nonneuropathic causes of pain, such as infection, musculoskeletal pain, or oncologic recurrence [8]. Lack of awareness in patients about treatment possibilities is a big factor that leads to prolonged suffering, and hence, management should involve proper education regarding common postmastectomy sequelae and the many available treatment options.

The different modalities of PMPS treatment described in the contemporary literature include psychological interventions, pharmacological interventions, interventional therapy, physical therapy, and surgery [71].

Psychological Interventions

Implementation of psychological interventions helps with chronic pain control as well as allows patients to effectively cope during this difficult phase of their lives [72]. Jassim et al. studied 28 randomized controlled trials involving 3940 breast cancer patients and evaluated the effectiveness of psychological interventions [73]. They found that psychological intervention, in particular cognitive behavioral therapy, produced highly favorable effects on anxiety, depression, and mood disturbance. In a meta-analysis by Haller et al., the effectiveness of mindfulness-based stress reduction was examined in 1709 breast cancer survivors, and a significant improvement was found in health-related quality of life, fatigue, sleep, stress, anxiety, and depression [74].

Pharmacological Therapy

A wide range of medications have been shown to be effective in PMPS, and an appropriate selection depends upon patient co-morbidities, drug adverse-effect profiles, potential long-term consequences, and cost-effectiveness. Various medication choices by class are briefly outlined below:

Antidepressants: Tricyclic antidepressants (TCAs) and serotonin-norepinephrine reuptake inhibitors (SNRIs) are commonly prescribed to treat PMPS at lower doses than those needed for anti-depressant effects. TCAs such as amitriptyline and nortriptyline inhibit the re-uptake of neurotransmitters including norepinephrine, serotonin, and adenosine at nerve endings, thereby increasing their concentration, which, by unclear mechanisms, plays a role in the analgesic effects [75].

Additionally, TCAs antagonize the N-methyl-D-aspartate (NMDA) receptors. In the context of pain, NMDA receptor activation is believed to contribute to the development of the wind-up phenomenon, wherein the intensity of pain increases over time for a given stimulus and causes central sensitization. NMDA receptor antagonism results in a decrease in sustained neuronal depolarization, thereby decreasing excitatory transmission along afferent pain pathways and resulting in the alleviation of pain. Larsson et al. systematically reviewed the treatment modalities and found that antidepressants lead to a statistically significant reduction of PMPS symptoms [17]. SNRIs, including duloxetine and venlafaxine, also inhibit neurotransmitter reuptake and have been explored with varying degrees of success in PMPS [76].

Gabapentinoids: Pregabalin and gabapentin function by modulating voltage-activated calcium channels, which inhibit nerve conduction in pain-transmitting neurons and central sensitization [77]. These drugs share structural similarities with the gamma-aminobutyric acid (GABA) neurotransmitter. They might also play a part in the central nervous system's activation of noradrenergic pain-inhibitory pathways. In a retrospective analysis of 89 PMPS patients, de Miguel-Jimeno et al. discovered that 80% of patients had successfully acquired a significant long-term reduction in pain [78]. However, 10% stopped receiving medication because of the negative effects.

Pregabalin has the added benefit of being almost completely absorbed in the body, unlike gabapentin, and hence, with about three times the absorption, it reaches peak blood concentrations within one hour after ingestion. Kaur et al. conducted an open-label, single-arm, prospective study to evaluate the efficacy of Pregabalin in relieving PMPS and found that it brought about significant reductions in pain (visual analog scale (VAS) scores; baseline 5.50 ± 1.197, end of one month 2.40 ± 1.430, end of two months 2.10 ± 1.370) and thereby significantly improved the QOL [79].

Topical medications: Topical medications are preferred by some patients to avoid the adverse events associated with oral pharmaceutical treatments. Of the various topical agents reported, capsaicin appears to be the most promising. Capsaicin is believed to act by leading to the attenuation of cutaneous hypersensitivity through a process described as "defunctionalization" [80].

Dupoiron et al. reported an important or complete effect for 70.7% and 56.0% of applications in patients with PMPS existing for less than 12 months and greater than 10 years, respectively [81]. The adverse effects noticed included application site reactions in 54.4% of patients (mainly burning sensation or pain, 45.9%, or erythema, 30.8%) and high blood pressure in 7.2%. Flother et al. reported pain relief without tolerance development with the application of capsaicin 8% patches in a 62-year-old female with intense PMPS that had persisted chronically over months and had not sufficiently responded to oral medication including tapentadol and pregabalin [82].

Opioids are the cornerstone of therapy for acute or chronic pain of moderate to severe intensity. They act on three types of opioid receptors: µ, δ, and κ (MOR, DOR, and KOR) in the central nervous system, but studies have suggested that these receptors also occur in other tissues, thereby creating a possibility for the topical use of opioids to achieve local analgesia without systemic adverse effects [83]. Accordingly, Mohamed et al. investigated the effect of topical morphine (in one of three doses: 5, 10, or 15 mg) and found that topical morphine controlled acute postmastectomy pain in a dose-dependent manner and reduced the incidence and severity of PMPS [84].

Interventional Therapy

Nerve blocks: The problematic nerves are identified by a thorough history and physical exam, and after real-time visualization under ultrasound, they are directly targeted for perineural injections with local anesthetics, steroids, or neurolytic substances such as phenol or alcohol. If the pain is not consistent with a specific nerve or otherwise appears to involve multiple nerves, various regional or interfascial plane blocks are used as options for pain relief.

Wijayasinghe et al. assessed the feasibility of intercostobrachial nerve (ICBN) blockade and its effects on pain and sensory function in 16 patients with PMPS [85]. They found that the second intercostal space could be visualized on ultrasound and that an ICBN block could be adequately performed, thereby achieving significant pain relief. Even the pre-existing areas of hypoesthesia decreased in most of the participants. In another study, Wijayasinghe et al. examined the effects of pectoral nerve block (Pecs block) on summed pain intensity (SPI) and sensory function (through quantitative sensory testing) in eight patients with PMPS [86]. The study suggested that the pectoral nerves play a role in the maintenance of PMPS and, hence, further research in the field was suggested. Takimoto et al. suggested that serratus plane block (SPB) has the potential to emerge as a treatment modality for PMPS as it can be performed more safely and easily than neuraxial approaches [87].

Neuromodulation: Neuromodulation attempts to change or disrupt the firing patterns of nerves via electrical stimulation. Dorsal root ganglion stimulation is one such modality that involves the placement of electrodes directly on the dorsal root ganglion and focused stimulation of the first-order neuron. Morgalla reported a case wherein the procedure was well tolerated and the pain score dropped from an 8/10 to a 4/10 for four years with a 50% reduction in the medication regimen [88]. However, the patient, due to local pain, required the transfer of the generator from the right gluteal region to the right abdominal region, but she was able to resume her job, and her functional status returned to baseline. Hetta et al. evaluated and compared the analgesic efficacy of pulsed radiofrequency (PRF) when delivered either on the thoracic dorsal root ganglion (DRG) of intercostobrachial nerves (thoracic DRG 2, 3, and 4) or their corresponding thoracic paravertebral nerves (PVNs) [89]. They enrolled 64 patients with PMPS for the study and concluded that PRF of both the thoracic DRG and the thoracic PVN are both effective treatments; however, PRF of the DRG provided a better long-term analgesic effect. They recommend that PRF of the DRG be reserved for cases that failed to gain adequate response to PRF of the thoracic PVN in conjunction with medical treatment due to the inherent risk of performing thoracic foraminal interventions and the technical difficulty of targeting the thoracic DRG.

Scrambler therapy is another neuromodulation modality based on non-invasive electrical neurocutaneous stimulation. The device generates 16 different types of waveforms that resemble action potentials and delivers them to the surface receptors of the c-fibers, thereby transmitting "non-pain" information along the damaged pathways to reduce central sensitization. In a series by Smith et al., patients achieved significant (over 75%) and sustained (months) reductions of allodynia, hyperalgesia, and pain, thereby reporting marked improvements in their QOL and functional status [90]. No participant reported any side effects.

Physical Therapy

The function of the upper extremities and shoulder can be hindered by chronic discomfort in the upper arm, axilla, and chest, which results in persistent stiffness, limited ROM, and frequently worsening pain. Physical and occupational therapy may therefore be involved. De Groef et al. reviewed the effectiveness of various postoperative physical therapy modalities and the timing of physical therapy after treatment of breast cancer on pain and impaired ROM of the upper limb [91]. They suggested that multifactorial physical therapy and active exercises were effective in treating pain and impaired ROM. However, they emphasized the need for more in-depth research in this area.

Surgical Interventions

Surgical treatment for PMPS is an option reserved for patients who exhaust other modalities and fail to show an adequate response. Surgical operations carry a potential risk of worsening the symptoms and, hence, before the adoption of this option, the possibility of failure to improve symptoms needs to be discussed as part of informed consent, and a viable backup plan must be kept in place should the operation fail.

Exploration 

The areas of maximal point tenderness are carefully marked. Exploration is performed, and scar tissue, a neuroma, or a mechanical obstruction (i.e., surgical clips or sutures) may be encountered. If a nerve is found entering the scar bed, then dissection is carried out until healthy nerve bundles are freed proximally and distally. Nerve ends are coapted, and if there are no suitable distal nerve targets, the proximal nerves are buried into non-scarred tissue. Alternatively, allogeneic nerve grafting to regional nerves, nerve conduits, or allograft nerves is conducted.

Hart et al. conducted a retrospective review of seven PMPS patients who had intercostal neurectomy procedures along with muscle or dermal wrapping of the resected nerve's proximal end [92]. With the help of local anesthetic nerve blocks, the suspected nerves were located, and each patient had an average of 3.14 neurectomies (with a range of 1-6). Following surgery, pain scores significantly decreased, and 86% of patients reported being pain-free or "considerably improved" on long-term follow-up. Broyles et al. retrospectively reviewed 10 patients with PMPS in whom affected intercostal nerves were identified with a diagnostic local anesthetic block and then dissected and implanted into local muscle [93]. Most of the patients experienced long-lasting relief from pain.

Fat Grafting

Recent research has outlined the advantages of fat grafting for PMPS patients with painful scar retraction and nerve entrapment. Juhl et al. conducted a controlled, randomized experiment to ascertain whether fat grafting had any analgesic effects on PMPS and whether it remodeled the mastectomy scar [94]. The study analyzed a total of 15 patients (fat grafted: 8, seven control: 7), and the average amount of grafted fat was 71 ± 24.6 mL. Fat grafting significantly decreased pain (55%), according to the study. Furthermore, health-related QOL and scar quality also showed statistically significant improvements. Maione et al. performed autologous fat grafting in 59 patients and compared the outcomes with those of a control group of 37 patients who underwent no additional surgical procedures [95]. A significant reduction in pain was detected in patients treated with autologous fat grafting, and it was concluded that due to its safety, efficacy, and optimal tolerability, the procedure can be considered useful in treating PMPS. Caviggioli et al. similarly came to a comparable conclusion after using autologous fat grafts to treat 72 PMPS patients [96]. However, Sollie et al. failed to find that fat grafting was in any way more effective than a placebo for treating PMPS [97].

Laser Therapy 

Recent years have seen a rise in the use of high-intensity laser therapy (HILT) as a treatment for pain of musculoskeletal origin [98]. The use of laser therapy is a particularly under-researched strategy, even within the relatively scant PMPS literature [69]. In a prospective study, Ebid and El-Sodany enrolled 61 women (30 in the laser group and 31 in the placebo laser group) to explore the potential utility of this tool in PMPS patients [99]. In contrast to the placebo laser group, which received placebo HILT plus routine physical therapy program, the laser group received HILT three times per week for four weeks. The laser group had a significant improvement in QOL outcomes, a decrease in pain scores, and a significant increase in shoulder ROM after four weeks of treatment and after 12 weeks of follow-up. Infrared laser and light-emitting diode (LED) were used in combination by Trelles and Calderhead for the treatment of PMPS in a 44-year-old female, and they reported reasonably encouraging results [100].

Relation of uncontrolled pain with patient outcomes and healthcare costs

Poorly controlled postoperative pain has been found to adversely impact patient outcomes, and increase the length of hospitalization and overall healthcare costs. Hence, there is a need to adopt a consistent, systematic, evidence-based, and holistic approach to acute pain management [101], and this shift would expectedly translate into reduction of PMPS in the long term.

Recent studies have suggested that chronic pain typically begins as acute poorly controlled postoperative pain, but gradually transitions into a persistent pain condition with neuropathic features that are unresponsive to opioids [102]. Research into the pathogenesis of this transition has led to a stronger appreciation of the use of more effective and safer analgesic regimens [103,104].

Uncontrolled pain has been found to trigger a complex neurohormonal cascade that negatively impacts nearly every organ system and results in renal and gastrointestinal dysfunction, increased incidence of infection, cardiopulmonary and thrombotic complications, impairment of wound healing, adverse psychological effects, and poorer functional recovery and QOL [105]. 

Algorithmic, Multidisciplinary Approach

The research on PMPS is still very limited and there is no unified line of action that may be projected as a gold standard. However, certain high-volume centers have devised their management protocols and published them in recent years for ease and possible adoption by healthcare providers. Yang et al. from Memorial Sloan Kettering Cancer Center, New York have proposed an algorithm to provide a stepwise application for selecting the appropriate therapies in the management of PMPS [106].

**Figure 3:**
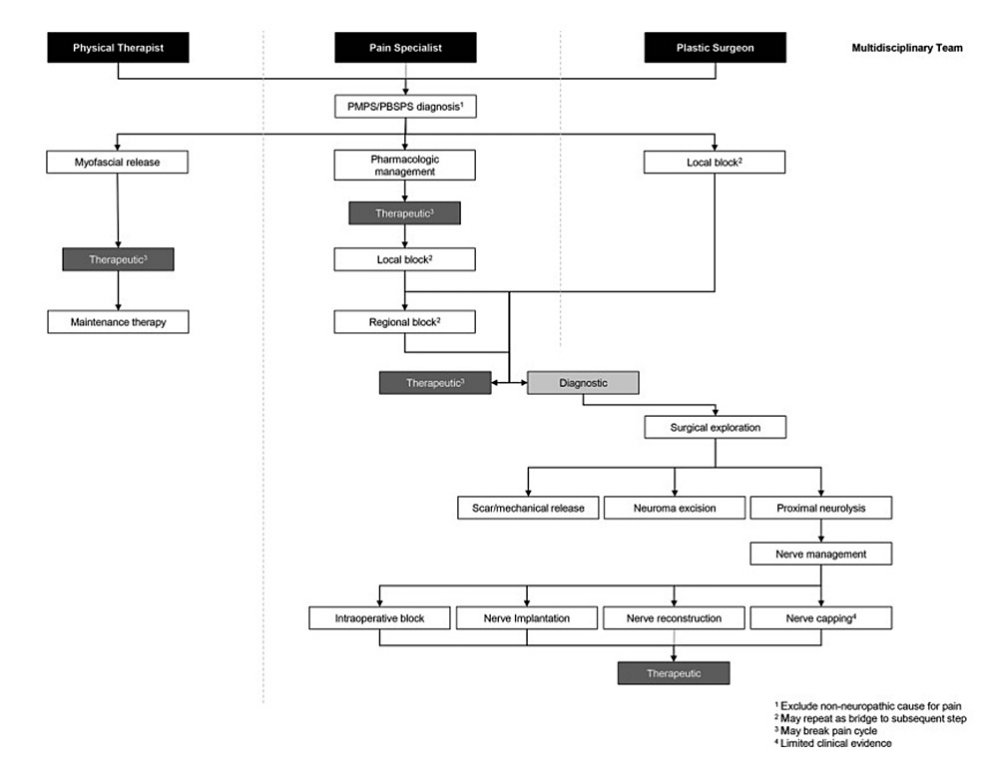
An algorithmic, multidisciplinary approach to the care of patients with PMPS. Image Source: Beederman and Bank, 2021 [8]; Open Access, Creative Commons Attribution-Non Commercial-No Derivatives License 4.0 (CCBY-NC-ND) PMPS: postmastectomy pain syndrome

Beederman and Bank from the University of Chicago Medicine, Chicago, have proposed an algorithm (Figure 3), in which stress was laid upon the creation of a proper multidisciplinary treatment team for the management of PMPS [8]. Their team comprised plastic surgeons, physical therapists, and pain specialists, and they showed that local blocks can be used both for diagnosis and for symptom management and can be performed either by the plastic surgeon or by the pain specialist. Surgical intervention has been mentioned as a last resort when other, less-invasive options have been attempted.

Limitations 

The authors have relied on articles whose full text has been made available as Open-Access or else on literature subscribed by Qassim University Library and Saudi Digital Library. Hence, there is a possibility that some articles of relevance may not have been analyzed while drafting this narrative review.

## Conclusions

In breast cancer survivors, PMPS is a fairly common but little-discussed complication. Intercostobrachial nerve-related neuropathy is the most commonly recognized pathogenesis. The disorder has a negative effect on QOL. Before the management of breast cancer is undertaken, a multidisciplinary team should thoroughly investigate a patient to reduce the risk factors and then formulate a logical treatment plan. If a patient ends up developing PMPS, a holistic plan of management should be chalked out. It is imperative that healthcare professionals remain updated and that patients are adequately informed about the available preventative and therapeutic choices.
